# Early Revelation of Leprosy in China by Sequential Antibody Analyses with LID-1 and PGL-I

**DOI:** 10.1155/2013/352689

**Published:** 2013-01-28

**Authors:** Pan Qiong-Hua, Zheng Zhong-Yi, Yang Jun, Wen Yan, Yuan Lian-Chao, Li Huan-Ying, Steven G. Reed, Malcolm S. Duthie

**Affiliations:** ^1^Leprosy Hospital, Kaiyuan, Honghe Prefecture, Yunnan Province 661600, China; ^2^Institute of Dermatology of Honghe Prefecture, Yunnan 661400, China; ^3^Yunnan Provincial CDC, Kunming, Yunnan 650022, China; ^4^Beijing Tropical Medicine Research Institute, Beijing Friendship Hospital-Affiliate of Capital University of Medical Sciences, 95 Yong An Road, Beijing 100050, China; ^5^Infectious Disease Research Institute (IDRI), Seattle, WA 98104, USA

## Abstract

Leprosy is a disabling chronic infection, with insidious onset that often evades early detection. In order to detect new leprosy cases in a timely manner, we conducted surveillance visits in some difficult-to-reach mountain areas in South West China where the disease is still prevalent. Our data confirm that Chinese multibacillary (MB) leprosy patients have strong antibody responses against *Mycobacterium leprae* antigens ND-O-BSA and LID-1. Contacts of clinically diagnosed patients were then monitored at regular intervals by both physical examinations and the laboratory determination of antibody responses in sera collected during these examinations. Elevations in antibody titers indicated the onset of MB leprosy in one of the contacts, and diagnosis was subsequently confirmed on physical examination. Our data indicate that rising antibody titers can be used as a trigger for physical examination or increased monitoring of particular individuals in order to provide early leprosy diagnosis.

## 1. Introduction

Leprosy is the clinical manifestation of infection with *Mycobacterium leprae*.  With a slow growth rate of division every 12-13 days at an optimum temperature of 30°C, *M. leprae* bacteria infect macrophages and Schwann cells [1]. Infection of Schwann cells predisposes infected individuals to nerve damage, rendering leprosy, a chronic disabling infection with insidious onset typically characterized by the early involvement of skin and peripheral nerves. The immune response of the leprosy patient shapes the clinical presentation [2]. The response can limit bacterial growth and dissemination, resulting in few localized skin lesions and the definition of such patients as paucibacillary (PB). In cases where the response does not control bacterial growth, high bacterial burden arises, infection becomes systemic and many disseminated lesions and significant nerve function impairment can be observed; these patients are defined as multibacillary (MB). If such cases are left untreated, leprosy can progress to disfigurement and disability.

Although early detection is recommended by World Health Organisation (WHO), diagnosis is currently achieved only upon the recognition of clinical symptoms by skilled clinicians [3]. As leprosy is usually associated with low socioeconomic development, patients often have limited access to medical care and evade early detection. Misdiagnosis is also common in clinics within large municipalities. Estimates are that patients are misdiagnosed an average of 2 times, resulting in a substantial amount of time passing from the onset of clinical signs to proper diagnosis and treatment [4–7]. These issues can have a devastating impact on the patient, as the longer a patient goes untreated the greater their chance of developing irreversible nerve damage [8–10]. In addition, untreated MB leprosy patients are likely transmitting *M. leprae *and inadvertently generating new cases.

Labor-intensive physical examination for skin and peripheral nerve involvement can be conducted at regular intervals (typically every few months and/or at annually) in recognized leprosy-endemic regions to detect suspected cases before advanced clinical symptoms and disabilities are established. While new cases do still arise from the general population, surveillance campaigns are often streamlined by focusing screening for possible preclinical infections in household contacts of diagnosed cases, as these individual, are at an elevated risk of developing leprosy [11–13]. The application of specific, simplified diagnostic methods within surveillance campaigns could expand the number of individuals analyzed or increase the frequency of analyses. For example, quantitative measurement of antibodies against the phenolic glycolipid (PGL)-I and leprosy IDRI diagnostic-(LID-) 1 antigens has previously been instructive for diagnosis of MB leprosy [14–16]. More importantly, antibody levels against either of these antigens have previously been reported to rise prior to the attainment of clinical diagnosis [16, 17].

Despite the pronouncement of the elimination of leprosy at a national level at the 15th International Leprosy Conference, Beijing 1998, leprosy remains prevalent in some of the difficult-to-access mountain regions of South West China [18, 19]. Although labor-intensive and logistically complicated, clinical surveillance teams visit residents in several of these regions on a regular basis. This program facilitates the relatively early recognition of patients and the prompt provision of multidrug therapy (MDT). In this study, we applied simple ELISA to sera collected from suspected leprosy cases in Honghe Prefecture, Yunnan Province, to quantify antibodies against the PGL-I and LID-1 antigens and evaluate the utility of these within active surveillance programs for leprosy. 

## 2. Materials and Methods

### 2.1. Subjects and Samples

Sera were obtained from patients with leprosy (MB and PB) or pulmonary tuberculosis (TB) and healthy household contacts (HHCs) of MB leprosy patients within a hyperendemic site in South West China. MB and PB leprosy patient sera used in this study were derived from recently diagnosed, previously untreated individuals (Table 1). Sera from healthy individuals residing in a leprosy nonendemic region served as additional controls (NC).

Within an active surveillance program, HHCs of 9 MB and 1 PB patients from a leprosy-endemic village (coded as A3-1*) were first assessed for leprosy by physical examination in early 2008. Additional samples were collected during a follow-up trip in February 2009 and when leprosy onset was suspected (i.e., the appearance of suspicious lesions and/or rising antibody responses). A third visit to reexamine the HHC was made in October 2009. 

### 2.2. Determining Antibody Responses by ELISA

Antibody responses were assessed by ELISA using the well-known glycolipid (ND-O-BSA) and the specific protein antigen (LID-1) [14–16]. Polysorp 96 well plates (Nunc, Rochester, NY) were coated with 1 *μ*g/mL recombinant protein or 200 ng/mL ND-O-BSA, the synthetically derived B-cell epitope of PGL-I conjugated to BSA, in bicarbonate buffer overnight at 4°C and blocked for 1 hr at room temperature with PBST with 1% BSA on a plate shaker. Serum was diluted in 0.1% BSA and added to each well. Plates were then incubated at room temperature for 2 hrs with shaking. Plates were washed with buffer only, and horseradish peroxidase-conjugated IgG or IgM (Rockland Immunochemicals, Gilbertsville, PA), diluted in 0.1% BSA, was added to each well and incubated at room temperature for 1 hr with shaking. After washing, plates were developed with peroxidase color substrate (Kirkegaard and Perry Laboratories, Gaithersburg, MD), and the reaction was quenched by the addition of 1 N H_2_SO_4_. The optical density of each well at was read 450 nm. 

### 2.3. Statistics

The *P* values were determined using the Student's *t*-test.

## 3. Results and Discussion

### 3.1. Antibody Responses within the Study Population (Yunnan Province)

**Figure 1:**
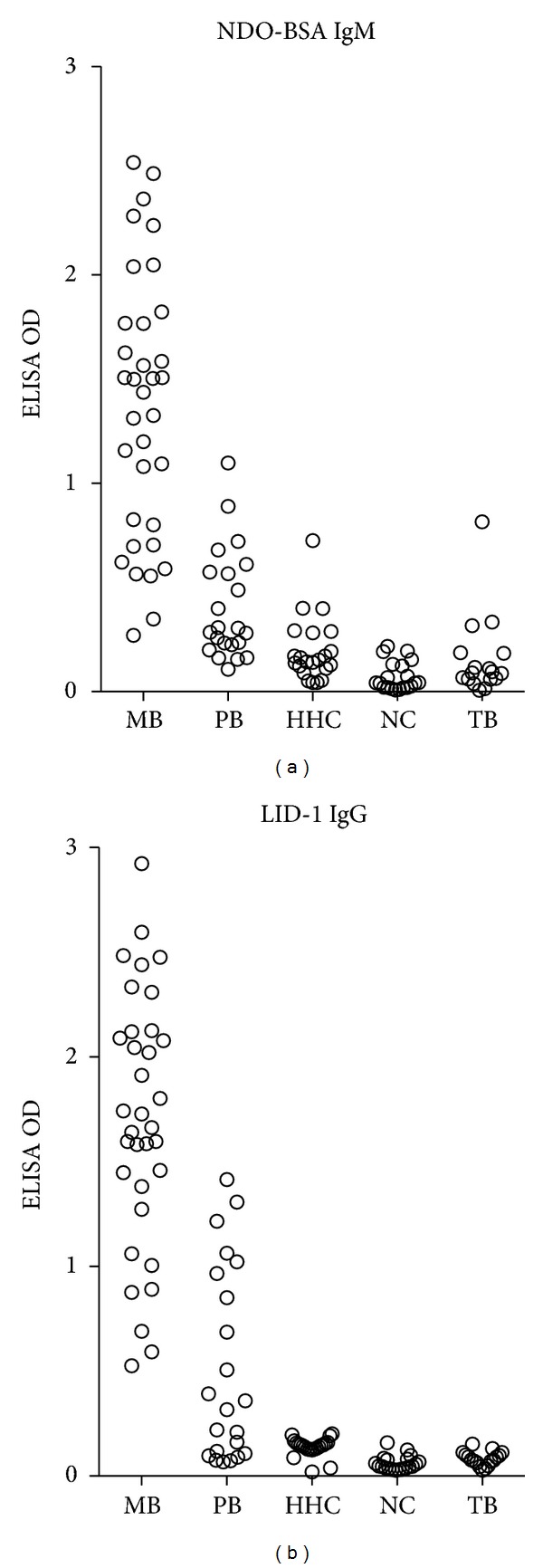
Antibody responses of leprosy patients in Yunnan Province. Sera from leprosy patients (MB = 34 and PB = 23), healthy household contacts of MB patients (HHC = 22), pulmonary tuberculosis patients (TB = 17), and uninfected controls (NC; 10 from Yunnan and 10 from Beijing) were assessed by ELISA against ND-O-BSA and LID-1. Glycolipid reactivity was assessed by IgM binding, and protein reactivity was assessed by IgG binding, with each sample distinguished by an individual marker.

ELISA was conducted against the ND-O-BSA and LID-1 antigens that have previously demonstrated the potential to diagnose (or confirm diagnosis of) leprosy. As previously reported, when compared with nondiseased individuals, MB patients had highly significantly elevated antibody titers against both ND-O-BSA and LID-1 (Figure 1 and Table 1). Although PB patients as a group could not be discriminated from the various control groups incorporated in the study (HHC, TB, and NC), a subset of PB patients did display elevated antibody titers (Figure 1). Similar results were obtained with ND-O-HSA (Table 1). These data indicate the utility of these antigens in detecting leprosy patients in China.

### 3.2. Prognostic Value of Antibody Analyses

**Figure 2:**
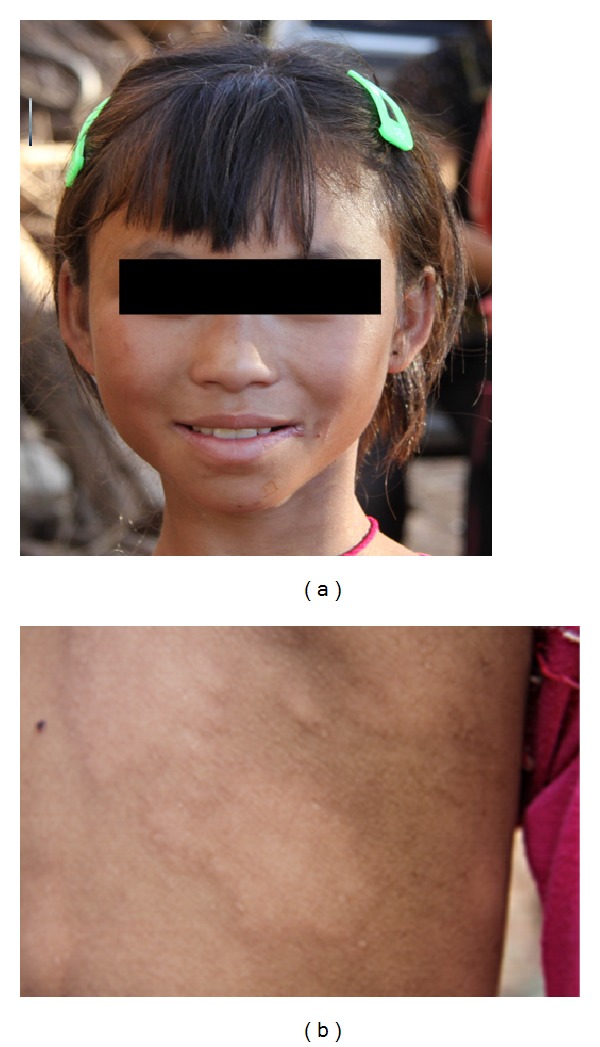
Physical presentation of leprosy in household contact C13. This 12-year-old female presented with elongated punched out lesions on her back (photograph taken in October 2009).

**Figure 3:**
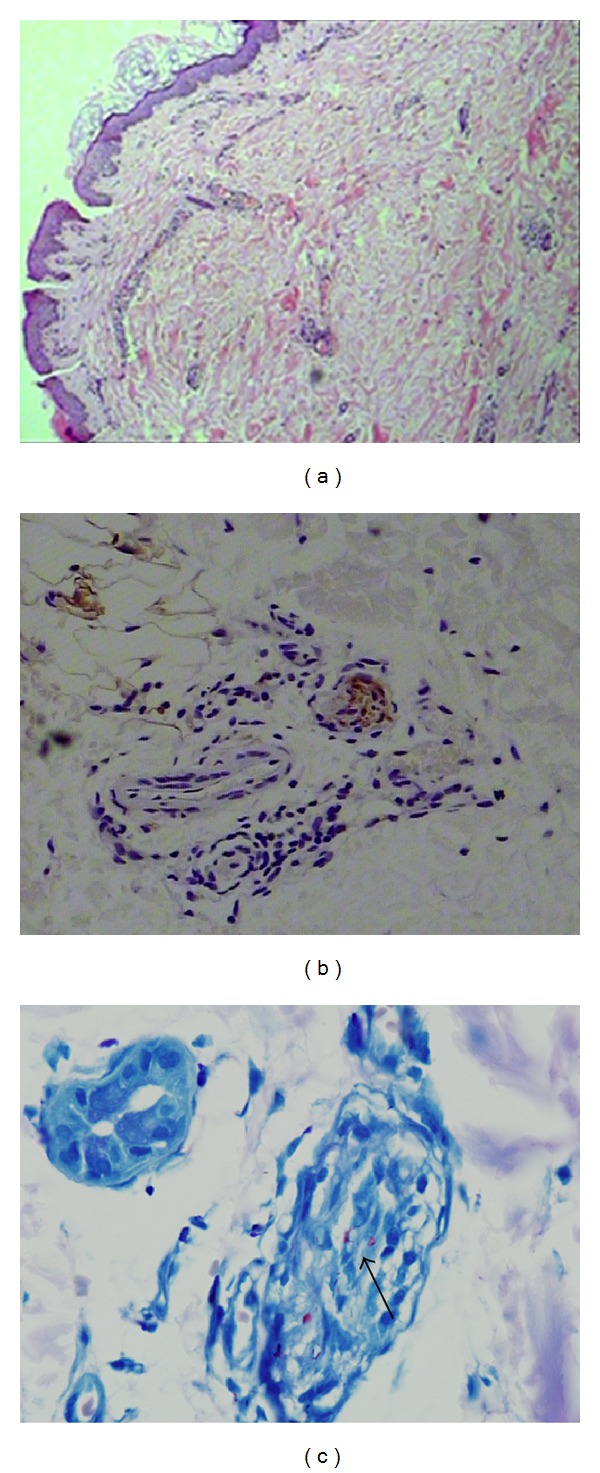
Microscopic presentation of leprosy in household contact C13. In (a), hematoxylin and eosin staining at ×200 magnification revealed epidermal atrophy, vascular dilation, and lymphocytic infiltration in neurovascular bundle. In (b), S-100 protein staining of a dermal nerve cross-section revealed perineural and perivascular infiltration (at ×400 magnification). In (c), single AFB in perineurium and aggregated AFB in fascicle could be observed at ×1000 magnification.

Sera collected during scheduled visits of leprosy patient households by the surveillance team were analyzed by ELISA. The rising antibody responses in the successive sera obtained from HHC-C13 indicated a potential onset of leprosy, alerting the monitoring team to return and examine this contact. During a physical checkup by the surveillance team, HHC-C13 appeared with numerous oval-shaped punched out lesions, with diminished sensation, of varying size in both the abdomen and the back (Figure 2). Upon histological examination of a biopsy collected from the edge of a back lesion, epidermal atrophy, vascular dilation, and lymphocytic infiltration could be observed in the neurovascular bundle (Figure 3(a)), and perineural and perivascular infiltration could be observed in the dermal nerve (Figure 3(b)). As expected, acid fast bacilli (AFB) were numerous, and the patient was determined to have a 3+ bacterial index (Figure 3(c)).

Two serum samples had already been obtained from this individual, and a third sample was collected at the time of clinical diagnosis of leprosy. Analysis of these sera revealed that IgM levels against ND-O-BSA rose between the second and third collections (Table 2). Even more strikingly, IgG levels against LID-1 were found to have risen between each collection.

The most likely source of *M. leprae* infection in this patient was her grandfather, who was a confirmed MB leprosy case with whom she shared a residence. It is noteworthy that he died in 2006, suggesting that this infection had been propagating for a minimum of 3 years. Although the laboratory data mitigated a clinical examination that led to the diagnosis of MB leprosy and provision of treatment, our data indicate that in this particular case, diagnosis based upon this antibody response could have been made, at a minimum, 8 months earlier than that achieved by clinical examination and before the appearance of such distinct symptoms. 

## 4. Conclusions

Regular clinical examinations of contacts of known leprosy cases are an effective, albeit labor-intensive, method of detecting new cases. Our data indicates that this process could be streamlined, and even expanded, by the use of serologic assays to screen this population. We suggest that rising antibody titers could be used as a trigger of fulminant physical examination or increased monitoring of particular individuals.

## Figures and Tables

**Table 1:** Comparison of antibody titers (mean and SD) of each group.

Group (*n*)	ND-O-BSA	ND-O-HSA	LID-1
	IgM	IgM	IgG
MB (34)	1.37 ± 0.64	1.79 ± 0.68	1.71 ± 0.60
PB (23)	0.41 ± 0.26	0.60 ± 0.38	0.51 ± 0.46
HHC (22)	0.20 ± 0.16	0.18 ± 0.17	0.14 ± 0.04
NC (20)	0.07 ± 0.07	0.13 ± 0.13	0.06 ± 0.03
TB (17)	0.16 ± 0.19	0.13 ± 0.15	0.08 ± 0.03

**Table 2:** Development of antibody responses over time in a contact that developed MB leprosy.

Time	ND-O-BSA	LID-1
	IgM	IgG
June 2008	0.338	0.298
February 2009	0.381	0.746
October 2009	0.948	1.406
